# Supplementary material for: S-acylation by ZDHHC20 targets ORAI1 channels to lipid rafts for efficient Ca2+ signaling by Jurkat T cell receptors at the immune synapse
Source: eLife. 2021 Dec 16;10:e72051. doi: 10.7554/eLife.72051 (PMC8683079; doi:10.7554/eLife.72051)

FULL SCANS Figure 1, Carreras-Sureda et al.

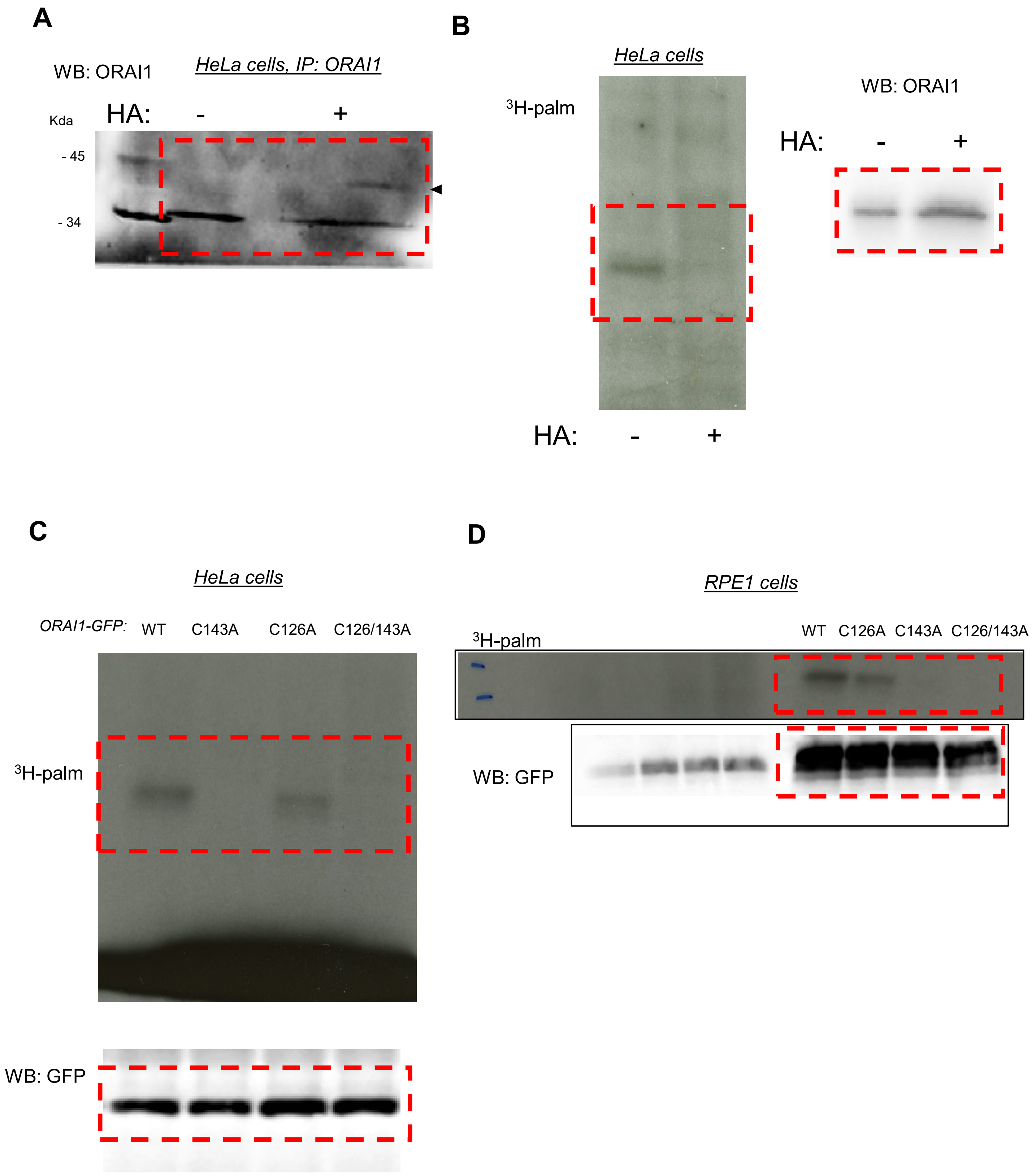

FULL SCANS Figure 4, Carreras-Sureda et al.

A

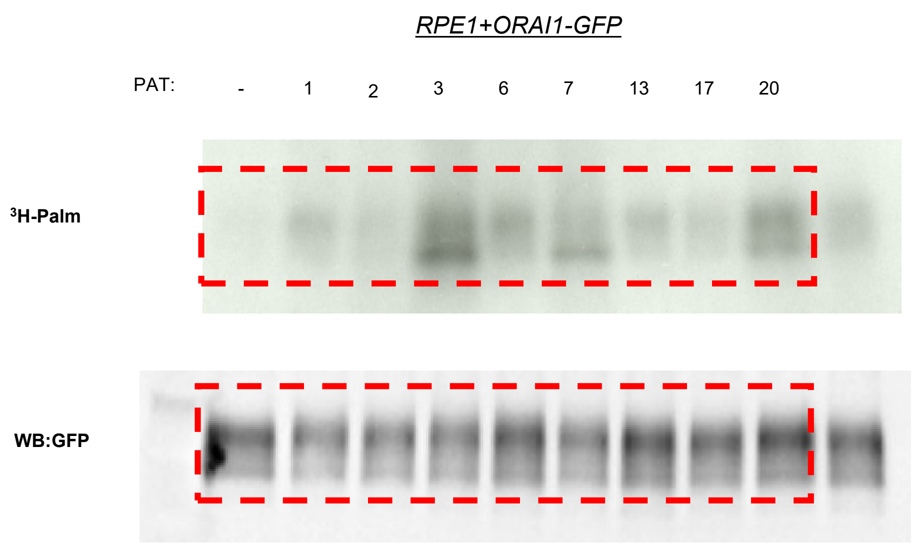

B

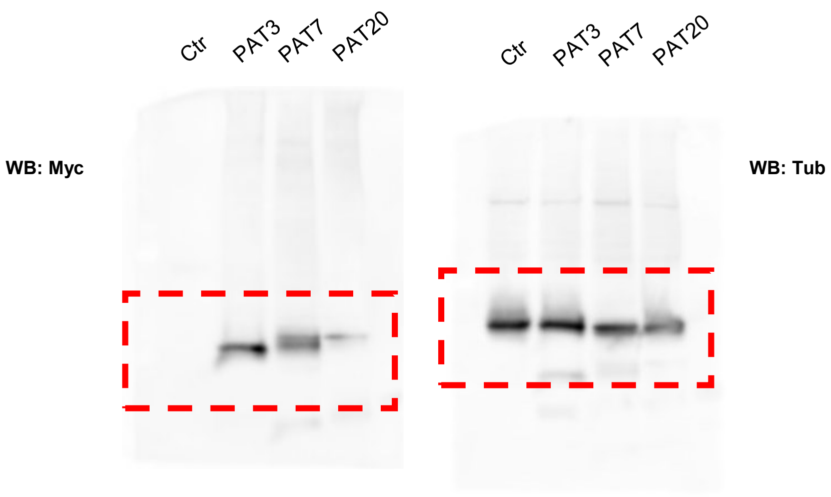

FULL SCANS Figure 5, Carreras-Sureda et al.

B

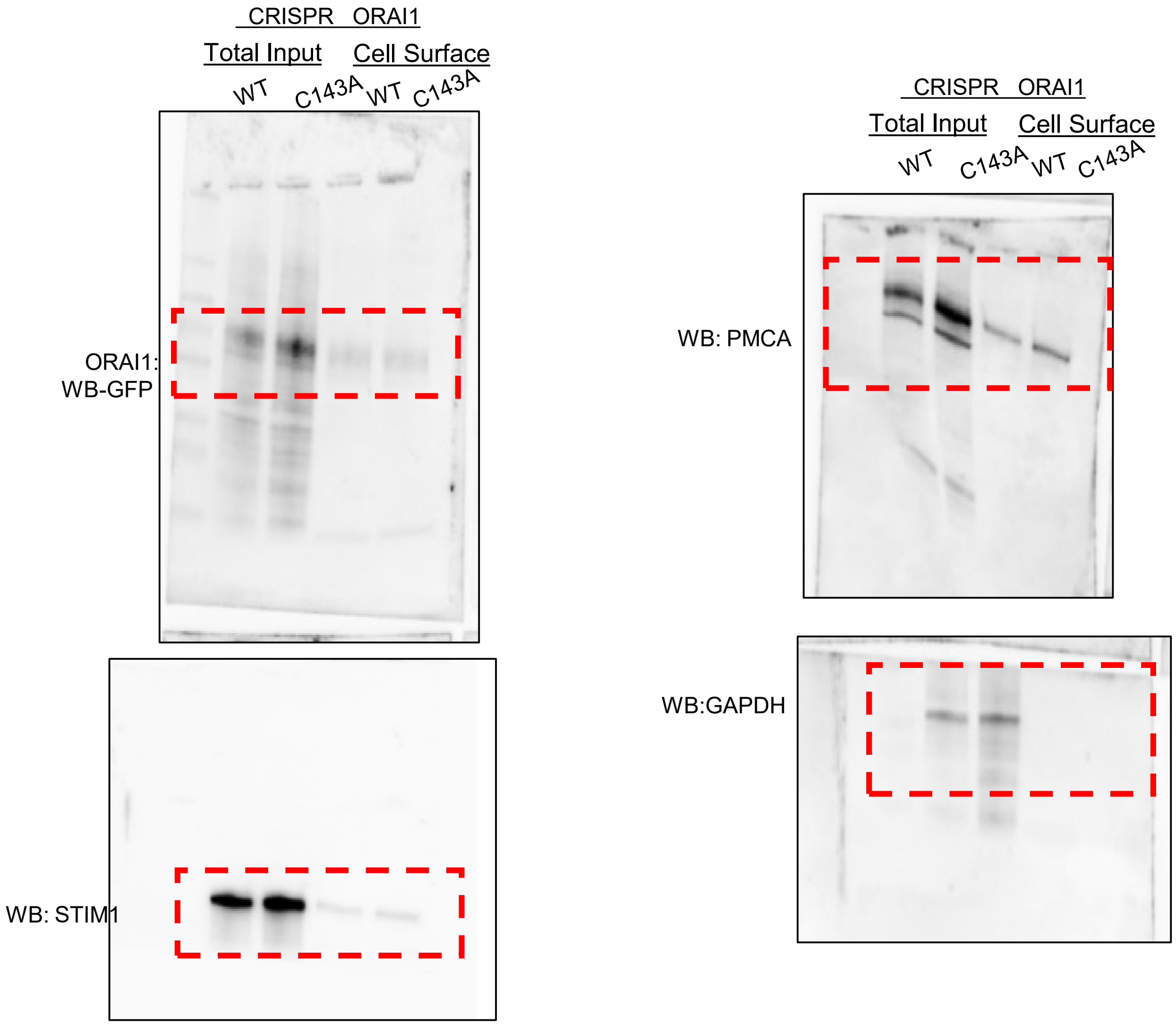

FULL SCANS Figure 5-supplementary 1

A

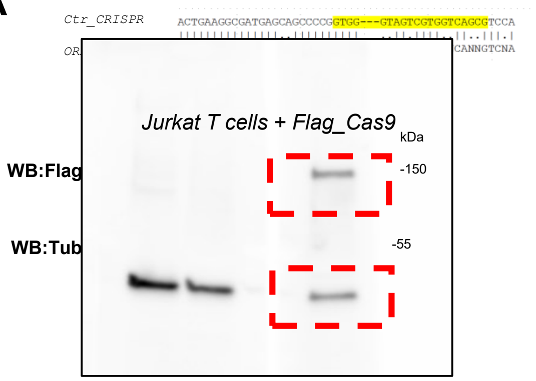

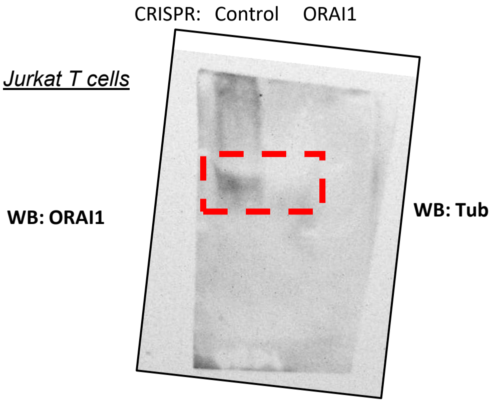

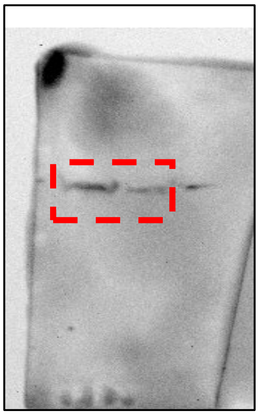

FULL SCANS Figure 5-supplementary 2

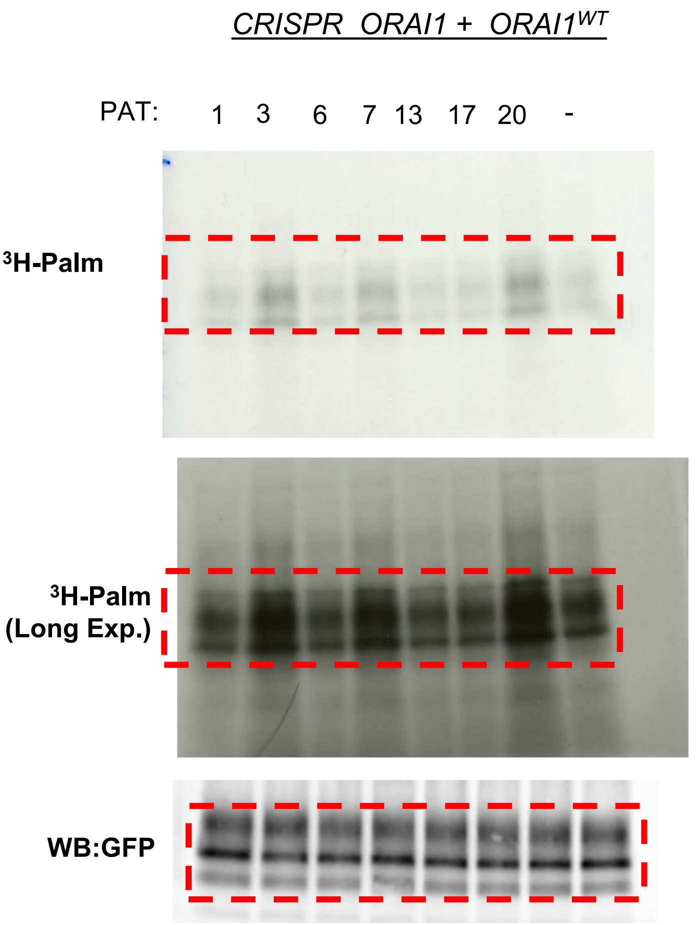

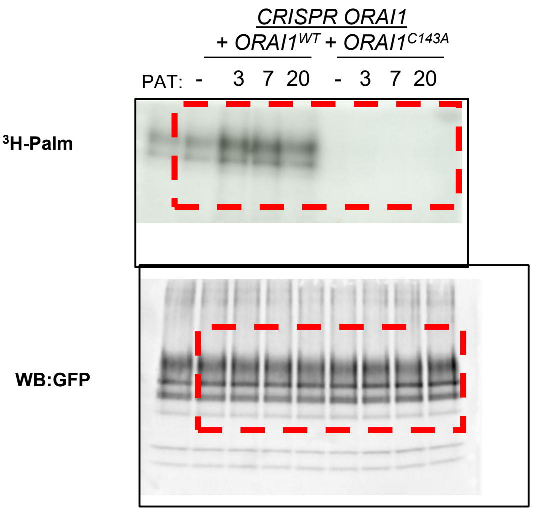

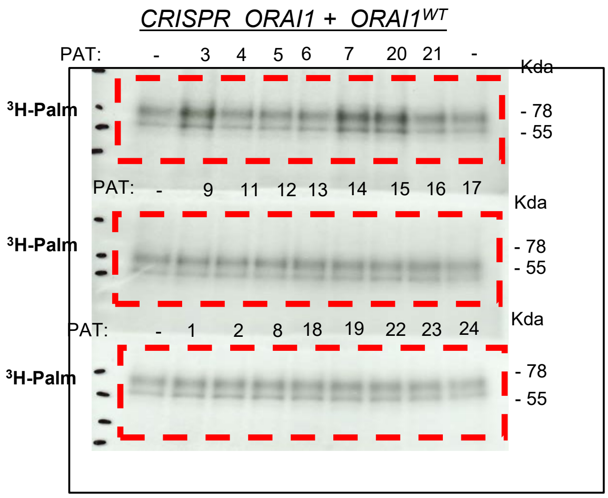

FULL SCANS Figure 6-supplementary 3

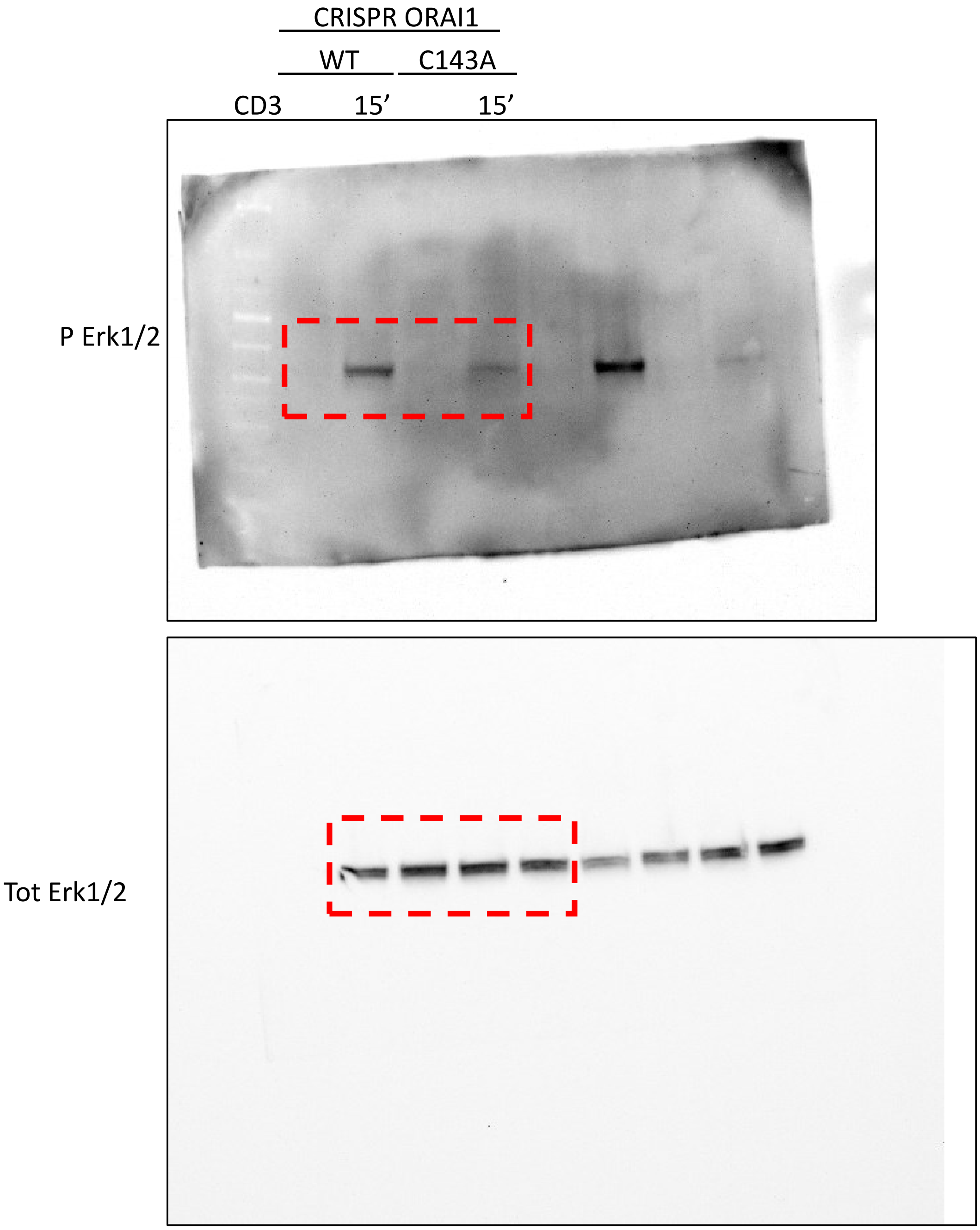

Supplement: Source data 2. — Red rectangles highlight the images used on the manuscript. [file elife-72051-supp2.pdf]
